# Shared Features Underlying Compact Genomes and Extreme Habitat Use in Chironomid Midges

**DOI:** 10.1093/gbe/evae086

**Published:** 2024-04-25

**Authors:** Lucas A Nell, Yi-Ming Weng, Joseph S Phillips, Jamieson C Botsch, K Riley Book, Árni Einarsson, Anthony R Ives, Sean D Schoville

**Affiliations:** Department of Integrative Biology, University of Wisconsin, Madison, WI 53706, USA; Department of Biology, Stanford University, Stanford, CA 94305, USA; Department of Entomology, University of Wisconsin, Madison, WI 53706, USA; McGuire Center for Lepidoptera & Biodiversity, Florida Museum of Natural History, University of Florida, Gainesville, FL 32611, USA; Department of Integrative Biology, University of Wisconsin, Madison, WI 53706, USA; Department of Biology, Creighton University, Omaha, NE 68178, USA; Department of Integrative Biology, University of Wisconsin, Madison, WI 53706, USA; North Central Agricultural Research Laboratory, USDA-ARS, Brookings, SD 57006, USA; Department of Integrative Biology, University of Wisconsin, Madison, WI 53706, USA; Mývatn Research Station, Skútustaðir IS-660, Iceland; Department of Integrative Biology, University of Wisconsin, Madison, WI 53706, USA; Department of Entomology, University of Wisconsin, Madison, WI 53706, USA

**Keywords:** extremophile, genome compaction, repeat elements, gene family evolution, positive selection

## Abstract

Nonbiting midges (family Chironomidae) are found throughout the world in a diverse array of aquatic and terrestrial habitats, can often tolerate harsh conditions such as hypoxia or desiccation, and have consistently compact genomes. Yet we know little about the shared molecular basis for these attributes and how they have evolved across the family. Here, we address these questions by first creating high-quality, annotated reference assemblies for *Tanytarsus gracilentus* (subfamily Chironominae, tribe Tanytarsini) and *Parochlus steinenii* (subfamily Podonominae). Using these and other publicly available assemblies, we created a time-calibrated phylogenomic tree for family Chironomidae with outgroups from order Diptera. We used this phylogeny to test for features associated with compact genomes, as well as examining patterns of gene family evolution and positive selection that may underlie chironomid habitat tolerances. Our results suggest that compact genomes evolved in the common ancestor of Chironomidae and Ceratopogonidae and that this occurred mainly through reductions in noncoding regions (introns, intergenic sequences, and repeat elements). Significantly expanded gene families in Chironomidae included biological processes that may relate to tolerance of stressful environments, such as temperature homeostasis, carbohydrate transport, melanization defense response, and trehalose transport. We identified several positively selected genes in Chironomidae, notably sulfonylurea receptor, CREB-binding protein, and protein kinase D. Our results improve our understanding of the evolution of small genomes and extreme habitat use in this widely distributed group.

**Significance**

Chironomid midges are known for having small genomes and tolerating many forms of environmental stress, yet little is known of the shared features of their genomes that may underlie these traits. We found that reductions in noncoding regions coincide with small chironomid genomes, and we identified duplicated and/or selected genes that may equip chironomids to tolerate harsh conditions. These results describe the key genomic changes in chironomid midges that may explain their ability to inhabit a range of extreme habitats across the world.

## Introduction

Non-biting midges of the family Chironomidae (order Diptera) are the most widely distributed group of freshwater insects (Armitage et al. 1995), with species found as far north as Ellesmere Island in Canada (Oliver and Corbet 1966); as far south as the Antarctic mainland (Usher and Edwards 1984); at 5,600 m above sea level in Himalayan glaciers (Kohshima 1984); and at 1,000 m below the surface of Lake Baikal in Siberia (Linevich 1963). Sediment in a productive freshwater river or lake is the archetypical chironomid habitat, but chironomids live in diverse environments such as ephemeral pools, hot springs, and water-filled cavities in plants, with even some marine or fully terrestrial species (Armitage et al. 1995). Chironomids may be ubiquitous because of their ability, as a group, to tolerate extreme conditions. Many forms of tolerance have been documented, including to low temperature (Lee et al. 2006; Rinehart et al. 2006), low oxygen (Burmester and Hankeln 2007), heavy metal contamination (Zheng et al. 2017), complete desiccation (Watanabe et al. 2002), and exposure to ionizing radiation (Gusev et al. 2010).

The extent to which shared genomic features underlie the extreme physiology and diverse habitat use of chironomids is largely unknown. The first sequenced chironomid genome was the Antarctic midge *Belgica antarctica*, one of the smallest recorded insect genomes (Kelley et al. 2014). The number of protein-coding genes in the *B. antarctica* genome was similar to other nonchironomid dipterans, whereas repeat elements and noncoding regions were reduced, and the authors concluded that the small genome size was likely an adaptation to its extreme environment. However, genome sizes were later estimated for 25 chironomid species by flow cytometry, showing that small genome size is likely an ancestral trait in chironomids and that smaller genomes within the family do not necessarily correlate with particularly stressful environments (Cornette et al. 2015). Additional reference assemblies have yielded further insights into individual types of stress tolerance, including hemoglobin gene repeats underlying copper tolerance in *Propsilocerus akamusi* (Sun et al. 2021) or a single chromosome in *Polypedilum vanderplanki* containing clusters of duplicated genes mediating desiccation tolerance (Gusev et al. 2014; Yoshida et al. 2022). Some commonalities have emerged, such as upregulation in antioxidant genes as a common mechanism for chironomid tolerances to heavy metal exposure, desiccation, and cold (Lopez-Martinez et al. 2008; Zheng et al. 2011; Gusev et al. 2014). Nonetheless, no study has explicitly compared chironomids to other dipterans to help understand genome evolution in this ubiquitous group.

Here, we generated two high-quality, annotated chironomid reference assemblies, a new assembly for *Tanytarsus gracilentus* (subfamily Chironominae, tribe Tanytarsini) and an improved assembly for *Parochlus steinenii* (subfamily Podonominae). We also constructed a time-calibrated phylogenomic tree for nine chironomids plus five dipteran outgroups. We used this phylogeny to inform analyses of (i) genome features associated with compact chironomid genomes and (ii) gene family evolution and positive selection that relate to extreme habitat use.

## Results and Discussion

### Genome Assemblies

We generated 23.08 Gb (∼243×) Oxford Nanopore Technologies (ONT) reads (read length N50 = 7,539 bp) and 22.11 Gb (∼232×) Illumina reads from our samples of *T. gracilentus*. The resulting assembly was 91.83 Mb in size, which closely matches the size estimated by back-mapping reads to the final assembly (95.10 Mb). We also created an assembly for *P. steinenii* based on sequencing reads from the Sequence Read Archive (SRA). This assembly's size of 143.57 Mb closely matches the published prediction of 143.8 Mb (Shin et al. 2019). These assemblies are two of the most contiguous gap-free chironomid assemblies to date (Table 1), although they are not arranged onto chromosomes as in *P. vanderplanki* and *P. akamusi* (Sun et al. 2021; Yoshida et al. 2022). Their high BUSCO (diptera_odb10 library) completeness percentages (*T. gracilentus* = 91.60% [90.53% single-copy, 1.07% duplicated], *P. steinenii* = 92.30% [90.99% single-copy, 1.31% duplicated]) indicate high quality assemblies. Both assemblies had negligible contamination from other species (maximum from sendsketch.sh: *T. gracilentus* = 0.02%, *P. steinenii* = 0.01%).

**Table 1:** Summary statistics for the final *T. gracilentus* and *P. steinenii* assemblies and for other assemblies of Chironomidae species available on GenBank. Percent complete was calculated using BUSCO complete genes based on the diptera_odb10 library.

Species	Total length (Mb)	Contigs	Contig N50 (bp)	GC (%)	Complete (%)	Accession
*Tanytarsus gracilentus*	91.83	45	7,014,169	32.4	91.57	GCA_038502055.1
*Chironomus riparius*	191.84	82	19,590,381	30.7	92.60	GCA_917627325.3
*Chironomus tentans*	213.46	64,545	7,697	31.2	89.35	GCA_000786525.1
*Polypedilum vanderplanki*	118.97	2,066	219,078	28.1	93.18	GCA_018290095.1
*Polypedilum pembai*	122.92	15,099	16,153	28.6	91.90	GCA_014622435.1
*Belgica antarctica*	89.58	22,152	13,687	38.9	91.72	GCA_000775305.1
*Clunio marinus*	85.49	24,952	154,800	31.8	93.18	GCA_900005825.1
*Propsilocerus akamusi*	85.84	144	6,207,813	33.8	92.79	GCA_018397935.1
*Parochlus steinenii*	143.57	55	7,709,542	31.0	92.27	GCA_038502155.1

### Genome Annotations

For *T. gracilentus*, we generated Illumina RNA-seq reads from adults (52.81 Gb, ∼555×) and juveniles (63.28 Gb, ∼665×). The total estimates of protein-coding genes for *T. gracilentus*, *P. steinenii*, and *Culicoides sonorensis* were similar to other chironomids and dipteran outgroups (supplementary table S1, Supplementary Material online). BUSCO completeness was high for the coding sequences from all sets of gene predictions, indicating good performance of the gene predictions, but the number of duplicated genes in *C. sonorensis* was high (supplementary fig. S1, Supplementary Material online), likely as a result of a redundant assembly (12.5% BUSCO complete + duplicate genes). We were able to functionally annotate most of the proteins for each species (*Chironomus riparius* = 12,249; *C. sonorensis* = 12,971; *P. steinenii* = 11,645; and *T. gracilentus* = 11,369) (supplementary table S1, Supplementary Material online).

### Phylogeny

**Fig. 1.**
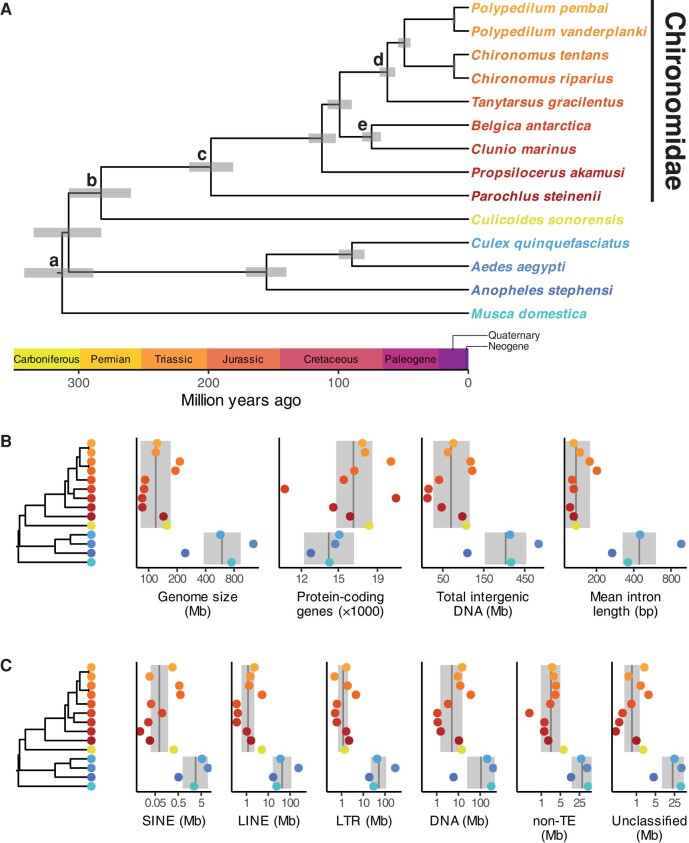
A) Time-calibrated phylogenomic tree showing relationships among chironomids and outgroups within order Diptera. Wide, gray bars indicate 95% credible intervals for node ages, and lowercase letters indicate fossil calibrations. Geological periods are shown above the *x*-axis. Species label colors and positions are the same as in the lower panels; chironomids are the top-most 9 species in shades of red/orange. B) Time-calibrated phylogeny next to genome size, number of protein-coding genes, total intergenic content, and mean intron length for all species in our phylogeny. C) Phylogeny alongside repeat content by class. B, C) Gray vertical lines indicate the mean estimates from the phylogenetic linear regressions for Chironomidae and Ceratopogonidae and for all other species. Gray envelopes indicate the 95% confidence interval bounds for these estimates computed via parametric bootstrapping. All measures are the log_10_-transformed totals across each species’ entire genome except for intron length, which is the mean of log_10_-transformed intron lengths.

To reconstruct the evolutionary history of chironomids, protein sequences from 1,436 diptera_odb10 single-copy, orthologous genes (935,062 total aligned sites after trimming) were used to construct the 14-species phylogenomic tree with dipteran outgroups. Matches to diptera_odb10 proteins and missing data from alignments were consistent across taxa (supplementary table S2, Supplementary Material online). All internal nodes had high bootstrap values (supplementary fig. S2, Supplementary Material online), and the topology of Chironomidae is consistent with previously published phylogenies (Cranston et al. 2012, 2010). MCMCTree results were consistent across runs, with the minimum pairwise correlation between mean posterior estimates being *r* = 1.000 and minimum effective sample size for any parameter being 559. Our deepest Chironomidae divergence times (Fig. 1A) are intermediate when compared to two published estimates (Cranston et al. 2012, 2010), but more nested splits, such as between *Propsilocerus* and the subfamilies Orthocladiinae and Chironominae, are similar to those in Cranston et al. (2010). The confidence intervals for our three deepest divergence events were overlapping, which is consistent with the wide range of published estimates for the divergence times between, for example, Chironomidae and Ceratopogonidae (137.3 to 296.9 Ma) (Bertone et al. 2008; Cranston et al. 2010, 2012; Rainford et al. 2014).

### Features Associated with Genome Size

**Fig. 2.**
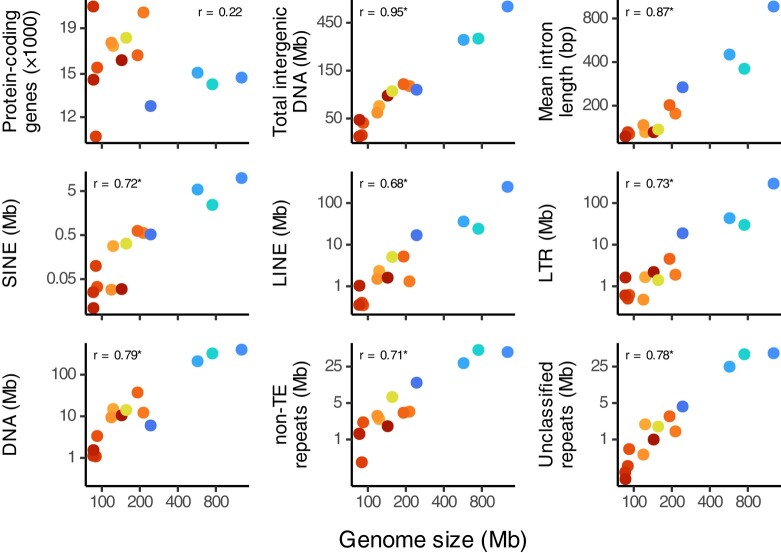
Genome size versus protein-coding genes, intergenic content, mean intron length, and repeat element content by class for dipterans in our phylogeny. Point colors and data transformations for all panels are as described in Fig. 1. Numbers in each panel indicate the estimate of the Pearson correlation coefficient between each variable and genome size using cor_phylo, and “*” indicates the correlation's 95% confidence interval did not overlap zero (supplementary table S3, Supplementary Material online).

To examine the drivers of genome size variation, we used a regression analysis to show that genome size, intergenic sequences, introns, and repeat elements all differed between nonchironomid dipterans and the group comprising Chironomidae and their closest relative, *C. sonorensis* (family Ceratopogonidae) (Fig. 1B and C). These same features were also significantly correlated with genome size across all species in our phylogeny (Fig. 2). In contrast, the number of protein-coding genes neither correlated with genome size nor differed between the families Chironomidae and Ceratopogonidae and other dipterans. These results suggest that the compact genomes of chironomids likely evolved in a common ancestor with Ceratopogonidae, although having only one species from Ceratopogonidae in our analysis makes this conclusion less certain. Our results also suggest that the reduction in genome size occurred through noncoding regions and repeat elements, which is consistent with a recent analysis of insect genome sizes (Cong et al. 2022). However, unlike Cong et al. (2022), our repeat element divergence landscapes reveal no obvious connection between repeat element ages and genome size (supplementary fig. S3, Supplementary Material online).

### Gene Family Evolution

**Fig. 3.**
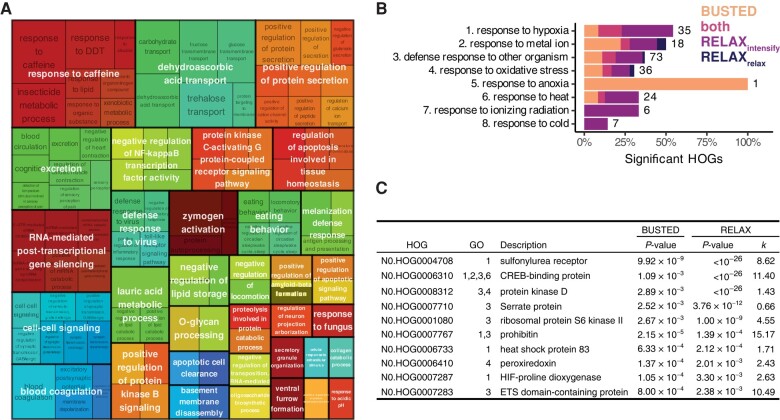
A) Treemap showing hierarchical structure of GO terms for HOGs that expanded significantly in Chironomidae. B) For each targeted GO term related to extreme physiology, we show the percent of single-copy HOGs that are significant for gene-wide positive selection (BUSTED), intensification of selection (RELAX_intensify_), relaxation of selection (RELAX_relax_), or both BUSTED and RELAX (either RELAX_intensify_ or RELAX_relax_). Numbers indicate total HOGs per GO term, and some GO terms share HOGs. C) List of HOGs with evidence for both positive selection and change in selection intensity in chironomids, in order of decreasing evidence for both tests (i.e. *P*_BUSTED_ × *P*_RELAX_). The GO column indicates the GO term(s) listed in B) associated with each HOG. Parameter *k* indicates the relative selection intensity for Chironomidae compared to outgroups (*k* > 1 means intensified selection, *k* < 1 means relaxed). HOG descriptions were extracted from the representative gene in *Culex quinquefasciatus*.

To examine gene families that evolved rapidly in chironomid lineages, we identified 19,878 total phylogenetic Hierarchical Orthologous Groups (HOGs) output from OrthoFinder and used CAFE for gene family evolution analysis. Of these, 9,057 HOGs were present at the root of the phylogeny, 883 changed significantly (*P* < 0.05) across the phylogeny, and nine had a significant change (*P* < 0.001) at the node separating Chironomidae from their nearest relative, *C. sonorensis* (supplementary table S4, Supplementary Material online). Gene Ontology (GO) terms overrepresented in these HOGs spanned a range of biological processes (Fig. 3A). Some of these may relate to tolerance of stressful or nutrient-limited environments, such as temperature homeostasis, carbohydrate transport, blood coagulation, and transport of dehydroascorbic acid (the oxidized form of vitamin A). Other overrepresented GO terms may indicate chironomids evolving in response to infectious agents (defense response to virus and response to fungus) and to plant chemical defenses (response to caffeine). Melanization defense response may relate to multiple adaptive pathways since it plays many physiological roles, including desiccation tolerance (Rajpurohit et al. 2008) and immune response (Nakhleh et al. 2017). Similarly, trehalose transport is likely a key stress-associated adaptation because of its diverse functions in cold and hypoxia tolerance (Elbein 2003; Chen and Haddad 2004), as well as protection from desiccation, as in the chironomid *P. vanderplanki* (Sakurai et al. 2008).

### Positive Selection

We tested for positive selection in 163 single-copy HOGs containing GO terms associated with tolerating stressful environments (Fig. 3B; supplementary table S5, Supplementary Material online). Using HyPhy's BUSTED method, 29 (17.8%) HOGs had evidence of gene-wide positive selection in chironomids compared to outgroups. Using HyPhy's RELAX method, 44 (27.0%) HOGs had evidence for relaxation or intensification of selection in chironomids, with most (40) of these indicating intensification. Ten (6.1%) HOGs were significant for both tests, only one of which had evidence for relaxed (instead of intensified) selection. Most of these HOGs were associated with “defense response to other organisms” (6) and/or “response to hypoxia” (5) GO terms. The genes with the strongest evidence for positive, intensified selection for Chironomidae were sulfonylurea receptor, histone acetyltransferase CREBBP, and protein kinase D. Sulfonylurea receptor is involved in chitin synthesis (Abo-Elghar et al. 2004) and protects against cardiac hypoxic stress in *Drosophila melanogaster* (Akasaka et al. 2006). CREB-binding protein is a lysine acetyl transferase that helps regulate a wide range of biological processes, including DNA repair (Cazzalini et al. 2014) and responses to hypoxia (Kung et al. 2004). Protein kinase D contributes to oxidative stress signaling and mediating of antioxidant enzyme expression (Storz and Toker 2003; Storz et al. 2005). These genes are interesting candidates for further study of the molecular basis of chironomid stress tolerance.

## Materials and Methods

Below is an overview of the methods, and more detailed information can be found in the Supplementary material online.

### Data Sources

In this study, we generated two new reference assemblies, three gene predictions, and four functional annotations. Reference assemblies and genome annotations for the comparative analysis (Tables 1 and supplementary table S6, Supplementary Material online) include previously published data, which were obtained from GenBank, SRA, VectorBase (Amos et al. 2022), or InsectBase (Mei et al. 2022).

### DNA Samples, Extractions, and Sequencing

We collected *T. gracilentus* from Lake Mývatn, Iceland (supplementary fig. S4A, Supplementary Material online). For long-read ONT sequencing, we extracted high-molecular-weight DNA from a single adult male (Quick 2018). ONT library preparation (EXP-PBC001 and LSK-109 kits) and sequencing (R9.4.1 FLO-MIN106 RevD SpotON flowcell on MinION II) were performed by the Roy J. Carver Biotechnology Center, University of Illinois Urbana-Champaign. We also used pooled short-read DNA-seq of adults for assembly polishing (as in Steward et al. 2021) and RNA-seq of both adults and juveniles to inform gene predictions (supplementary fig. S4B, Supplementary Material online). Extractions (using QIAGEN QIAcube HT and RNeasy Mini Kit), library preparation (using Celero PCR Workflow with Enzymatic Fragmentation and Illumina TruSeq Stranded mRNA), and sequencing were conducted by the University of Wisconsin–Madison Biotechnology Center.

### Genome Assemblies

For our *T. gracilentus* assembly, we first generated multiple assemblies from ONT reads using different assemblers (NECAT, SMARTdenovo, NextDenovo, and Flye) and then combined them into a single best assembly using quickmerge (supplementary tables S7 and S8 and fig. S5, Supplementary Material online). For each step of the assembly, we used BUSCO with the diptera_odb10 dataset to evaluate genome completeness and a custom Python script to evaluate contiguity. We estimated the genome size by mapping ONT reads back onto the final assembly using backmap. We generated the assembly for *P. steinenii* using NextDenovo on publicly available ONT sequences (SRA accessions: SRR8180978, SRR3951280, SRR3951285, SRR3951284, and SRR3951283). We looked for contamination in both final assemblies using unique 31-mers via sendsketch.sh from bbmap.

### Repeat Elements and Genome Annotations

We described repeat elements for all species by combining a de novo library of repetitive elements for each using RepeatModeler with a library of dipteran repeats from RepBase. We used RepeatMasker to summarize repeat elements by class and to calculate repeat element divergences. We used BRAKER and the GeneMark-ES Suite to create two sets of gene predictions, one using RNA-seq reads and another using the OrthoDB arthropod protein database. We combined them using TSEBRA. We functionally annotated genes using mantis that compares protein sequences to Pfam, KOfam, eggNOG, NCBI's protein family models, and the transporter classification databases.

### Phylogeny Construction and Finding Orthogroups

To construct the phylogeny, we first extracted amino acid sequences for single-copy orthologs from the diptera_odb10 database using BUSCO. We aligned sequences using MAFFT and then trimmed alignments using trimAl. Next, we partitioned by gene and used ModelTest-NG to optimize the substitution model for each partition. RAxML-NG was then used to generate a maximum likelihood (ML) tree and quantify bootstrapped branch support. We created a time-calibrated tree by combining the ML tree with fossil data from paleobiodb.org (queried on July 5, 2022) and previous time estimates using MCMCTree. We defined five calibration points: (i) 238.5 Ma minimum and 295.4 Ma maximum for the root (Benton et al. 2009), (ii) 242.0 Ma minimum for the superfamily Chironomoidea (Lukashevich et al. 2010), (iii) 201.3 Ma minimum for family Chironomidae (Krzeminski and Jarzembowski 1999), (iv) 93.5 Ma minimum for subfamily Chironominae (Giłka et al. 2022), and (v) 33.9 Ma minimum for the portion of subfamily Orthocladiinae containing genera *Belgica* and *Clunio* (Zelentsov et al. 2012). These estimates informed the parameters for the divergence time sampling distributions in MCMCTree (see supplementary table S9, Supplementary Material online for specific details). We used CODEML to inform the overall substitution rate and the “data-driven birth–death” method (Tao et al. 2021) to inform priors for the speciation birth–death process. ModelFinder was used to merge partitions (for better convergence in MCMCTree) and find the best model per partition.

We used OrthoFinder to identify phylogenetic HOGs. We input each species’ protein sets (filtering for the longest isoform per gene) and the time-calibrated species tree into OrthoFinder and used the set of HOGs for the root of the phylogeny for downstream analyses. We removed *Clunio marinus* from any analyses using HOGs because only 58.5% of its genes were assigned to orthogroups.

### Features Associated with Genome Size

We looked for associations between genome size and four genomic features in chironomids: protein-coding genes, intergenic sequences, introns, and repeat elements. We first tested for significant differences in each feature (including genome size) between chironomids and other dipterans to assess whether any associations likely pertain to chironomids specifically. For these tests, we grouped *C. sonorensis* (family Ceratopogonidae) with chironomids because doing so improved the log-likelihoods of our models. We used phylogenetic linear regressions via the R package phylolm (Ho and Ane 2014). We next tested for whether each feature was correlated with genome size to ascertain whether any changes that occurred likely contributed to genome compaction using the cor_phylo function in the phyr package (Li et al. 2020) that accounts for phylogenetic covariance.

### Gene Family Evolution

We used CAFE to identify HOGs that significantly expanded (*P* < 0.001) at the node separating Chironomidae from their most recent common ancestor. We used the enricher function in clusterProfiler to find enriched GO terms in our set of HOGs. We then used rrvgo to reduce the redundancy of the set of enriched GOs and to generate a treemap.

### Positive Selection

We used HyPhy to test for positive selection in Chironomidae in single-copy HOGs that were associated with an a priori list of GO terms associated with tolerance to extreme habitats (supplementary table S5, Supplementary Material online). We labeled Chironomidae on our tree to define our foreground branches, and then for each HOG, we tested (i) whether positive selection occurred for any chironomids using HyPhy's BUSTED method (Murrell et al. 2015) and (ii) whether selection intensified or relaxed for chironomids using HyPhy's RELAX method (Wertheim et al. 2015). We corrected *P*-values for multiple comparisons using the Benjamini–Yekutieli procedure.

## Supplementary Material

evae086_Supplementary_Data

## Data Availability

All reference assemblies and raw sequencing data are archived on NCBI under BioProject number PRJNA1044157. Data resulting from analyses (including annotations) are on Zenodo at https://dx.doi.org/10.5281/zenodo.10909791, and all scripts to run the analyses are archived on Zenodo at https://dx.doi.org/10.5281/zenodo.10909946.
