# Supplementary material for: Shared Features Underlying Compact Genomes and Extreme Habitat Use in Chironomid Midges
Source: Genome Biol Evol. 2024 Apr 25;16(5):evae086. doi: 10.1093/gbe/evae086 (PMC11076180; doi:10.1093/gbe/evae086)
Supplement: evae086_Supplementary_Data [file evae086_supplementary_data.pdf]

Supplementary Materials

Shared features underlying compact genomes and extreme habitat use in chironomid midges

Lucas A. Nell*, Yi-Ming Weng, Joseph S. Phillips, Jamieson C. Botsch, K. Riley Book, Árni Einarsson, Anthony R. Ives, Sean D. Schoville

* Corresponding author email: lucnell@gmail.com

Supplementary Materials and Methods

The full dataset we analyzed included 14 total species from order Diptera, nine of which were from family Chironomidae (table 1). Outside Chironomidae but within infraorder Culicomorpha, we included one species from family Ceratopogonidae (*Culicoides sonorensis*) and three from family Culicidae: *Anopheles stephensi*, *Aedes aegypti*, and *Culex quinquefasciatus*. To more fully represent order Diptera, we also included *Musca domestica*, which is part of suborder Brachycera, family Muscidae. Although more species from taxa closely related to Chironomidae, such as Ceratopogonidae, would improve the power of our analyses, we were limited by the scarce genomic resources for these groups. We gathered or created reference assemblies, gene predictions, functional annotations, and repeat libraries for all 14 species. Of these species, we generated new (1) sequencing reads for one (*Tanytarsus gracilentus*), (2) reference assemblies for two (*Parochlus steinenii* and *T. gracilentus*), (3) gene predictions for three (*C. sonorensis*, *P. steinenii*, and *T. gracilentus*), (4) functional annotations for four

(*Chironomus riparius*, *C. sonorensis*, *P. steinenii*, and *T. gracilentus*), and (5) repeat libraries for all species. The data we did not generate ourselves we collected from GenBank, Sequence Read Archive (SRA), VectorBase (Amos et al. 2022; Giraldo-Calderón et al. 2015), or InsectBase (Mei et al. 2022). In the next sections, we describe our data collection in order of use, from sample collection to functional annotations. We then describe how we used these data to construct a time-calibrated phylogeny and analyze genomic features associated with compact genomes and extreme physiology in Chironomidae.

***Tanytarsus gracilentus* samples, extractions, and sequencing**

We collected all *Tanytarsus gracilentus* from sites at Lake Mývatn, Iceland (65.5852º, −16.9816º) (fig. S4). DNA samples were stored in 95% ethanol and RNA samples in RNAlater, and all were kept at −20ºC in Iceland. During and after transport to the United States, DNA samples were stored at −80ºC, while RNA samples remained at −20ºC until extraction.

For long-read Oxford Nanopore Technologies (ONT) sequencing, we extracted high-molecular-weight DNA from a single adult male using a previously published protocol (Quick 2018). ONT library preparation and sequencing were performed by the Roy J. Carver Biotechnology Center, University of Illinois Urbana-Champaign. They used 50ng size-selected DNA to create a PCR library with the EXP-PBC001 and LSK-109 kits (Oxford Nanopore Technologies, Oxford, UK) and sequenced the library on one R9.4.1 FLO-MIN106 RevD SpotON flowcell for 72 hours on a MinION II sequencer.

We also used pooled short-read DNAseq for assembly polishing (Steward et al. 2021) and RNAseq to inform gene predictions. Extractions, library preparation, and sequencing were conducted by the University of Wisconsin–Madison Biotechnology Center, with sequencing on

an Illumina NovaSeq 6000 (2 × 150bp reads on an S4 flow cell). DNA was extracted from a pool of 7 adult males using a QIAcube HT from QIAGEN (Hilden, Germany), and DNA concentration was verified using the Qubit dsDNA HS Assay Kit (Life Technologies, Grand Island, NY). The sample was then prepared according to the Celero PCR Workflow with Enzymatic Fragmentation (Tecan Genomics, Redwood City, CA). Quality and quantity of the finished library was assessed using an Agilent TapeStation (Agilent, Santa Clara, CA) and Qubit dsDNA HS Assay Kit, respectively. DNAseq libraries were sequenced at ≥ 100× coverage. Two RNAseq libraries were also generated, one from a pool of 10 adult males and another from a pool of 155 first-instar juveniles. Samples were homogenized using a QIAGEN TissueLyser, then RNA was extracted using organic extractions followed by on-column DNase treatment with QIAGEN RNeasy Mini Kit. RNA quality and quantity were assessed using a NanoDrop (Thermo Fisher Scientific, Waltham, MA) and Agilent BioAnalyzer. Libraries were created using the Illumina TruSeq Stranded mRNA library preparation kit and were sequenced to obtain ≥ 100 million reads per sample.

Tanytarsus gracilentus genome assembly

We performed ONT basecalling and adapter trimming using Guppy v5.0.11 and the dna_r9.4.1_450bps_hac.cfg model. For both DNAseq and RNAseq Illumina reads, we used fastp v0.23.2 (Chen et al. 2018) for adapter trimming, polyG tail trimming, and correction in overlapping regions of paired reads. For RNAseq reads, we also used fastp to remove polyA tails, and we followed previous recommendations for mRNA data (MacManes 2014) by applying modest quality trimming ($Q \geq 5$) and a read length filter (≥ 50 bp). For mapping reads to

assemblies throughout, we used minimap2 v2.24 (Li 2018) for ONT reads, BWA-MEM v0.7.17 (Li 2013) for Illumina DNAseq, and HISAT2 v2.2.1 (Kim et al. 2019) Illumina RNAseq.

Our assembly strategy for *T. gracilentus* consisted of using multiple assemblers, then combining them into a single best assembly using quickmerge v0.3 (Chakraborty et al. 2016) (fig. S5, table S7). For each step of the assembly, we used BUSCO v5.4.3 (Manni et al. 2021) with the diptera_odb10 dataset (Kriventseva et al. 2019) to evaluate genome completeness and a custom python script to evaluate contiguity. To generate initial assemblies from ONT reads, we used NECAT v0.0.1 (Chen et al. 2021), NextDenovo v2.5.0 (Hu et al. 2023), SMARTdenovo v1.0.0 (Liu et al. 2021), and Flye v2.9 (Kolmogorov et al. 2019). Each was followed by ONT polishing: Racon v1.5.0 (3 rounds) (Vaser et al. 2017) then medaka v1.6.0 (https://github.com/nanoporetech/medaka). We assessed assemblies for evidence of heterozygosity-induced duplicate regions via the hist_plot.py script in purge_dups v1.2.5 (Guan et al. 2020), and then used purge_dups to remove repeated regions. We next merged every pairwise permutation of these assemblies using quickmerge. The four best permutations were used in a second round of merges that produced a single best assembly (table S8). We polished this assembly again with ONT reads using medaka, then polished it using pooled Illumina DNAseq reads (with uncalled bases removed using fastp) using NextPolish v1.4.0 (Hu et al. 2020). In addition to evaluating the final assembly completeness using BUSCO, we estimated the genome size by mapping ONT reads back onto the final assembly using backmap v0.5 (Pfenninger et al. 2022) and looked for contamination in the final assembly using sendsketch.sh (default settings) from bbmap v38.96 (Bushnell 2014).

Parochlus steinenii reads and genome assembly

We generated a new reference assembly for *Parochlus steinenii* based on previously published sequencing data (Shin et al. 2019). This species is especially useful because of its place within the subfamily Podonominae, which represents an outgroup with respect to *T. gracilentus* and the other chironomid species with reference assemblies. We used ONT reads (SRA accession: SRR8180978) and paired-end Illumina DNAseq reads (SRR3951280) for genome assembly, and paired-end Illumina RNAseq reads (SRR3951285, SRR3951284, and SRR3951283) for the annotation. Trimming of Illumina reads followed the methods described for *T. gracilentus*. We generated an initial assembly based on ONT reads using NextDenovo v2.5.0. We polished the assembly using 6 rounds of Racon with ONT reads, then using 6 rounds of NextPolish with Illumina DNAseq reads.

Culicoides sonorensis RNAseq reads

As a member of family Ceratopogonidae, the most closely related family to Chironomidae, *Culicoides sonorensis* is especially informative for understanding the evolutionary history of Chironomidae. To generate gene predictions for *C. sonorensis*, we used paired-end Illumina RNAseq reads from a project designed to boost gene expression under many different environmental conditions (Morales-Hojas et al. 2018). We accessed these reads from the SRA using the following accession codes: ERR637904, ERR637905, ERR637906, ERR637907, ERR637908, ERR637909, ERR637910, ERR637911, ERR637912, ERR637913, and ERR637914.

Repeat elements

We described repeat elements for all 14 species' assemblies by first creating a de novo library of repetitive elements for each using RepeatModeler v2.0.3 (Flynn et al. 2020) with the LTR structural finder enabled, then identifying many of the unclassified elements using TEClass v2.1.3 (Abrusán et al. 2009). Next, we combined this custom library with dipteran repeats from the RepBase RepeatMasker edition library v20181026 (Bao et al. 2015). We then soft-masked the reference assembly based on this combined library using RepeatMasker v4.1.2 (Smit et al. 2013). In the species for which we created new gene predictions, we used this soft-masked assembly for all analyses downstream, but for the others, we continued to use the original assemblies that were already soft masked. For all species, we used the annotation (*.out) file from RepeatMasker to summarize repeat elements by class. Next, we calculated repeat element divergences (CpG-adjusted Kimura substitution level) using RepeatMasker's calcDivergenceFromAlign.pl script that takes a RepeatMasker align file as input. We manually parsed the resulting divsum files to create repeat landscape plots.

Gene predictions and functional annotations

For the *T. gracilentus*, *P. steinenii*, and *C. sonorensis* genome annotations, we used BRAKER v2.1.6 (Brůna et al. 2021) and the GeneMark-ES Suite v4.71 to create two sets of gene predictions. The first set was based on RNAseq reads using GeneMark-ET (Lomsadze et al. 2014) and AUGUSTUS v3.4.0 (Stanke et al. 2008). The second set was based on the arthropod protein database from OrthoDB v10 using ProtHint v2.6.0, GeneMark-EP (Brůna et al. 2020), and AUGUSTUS. These two sets of predictions were combined to create a single consensus gene set using TSEBRA v1.0.3 (Gabriel et al. 2021) with a preference for genes predicted by

RNAseq by using the provided pref_braker1.cfg configuration file. We ran BUSCO on the CDS of the annotations to assess performance of the gene predictions. We functionally annotated genes for *Chironomus riparius,* as well as *T. gracilentus, P. steinenii,* and *C. sonorensis,* using mantis v1.5.5 (Queirós et al. 2021). This program uses DIAMOND v2.0.15 (Buchfink et al. 2021) and HMMER v3.3.2 (hmmer.org) to compare protein sequences to five different databases: Pfam (El-Gebali et al. 2019), KOfam (Aramaki et al. 2020), eggNOG (Huerta-Cepas et al. 2019), NCBI's protein family models (NPFM) (Lu et al. 2020), and the Transporter classification database (TCDB) (Saier et al. 2021). Databases were accessed on 12 July 2022.

Other data sources

We used reference assemblies from GenBank for seven chironomids (table 1). We also included assemblies from outgroups in families Ceratopogonidae (*Culicoides sonorensis*), Culicidae (*Anopheles stephensi*, *Aedes aegypti*, and *Culex quinquefasciatus*), and Muscidae (*Musca domestica*) (table S6). To find gene predictions and functional annotations for all species except those described in previous sections, we used GenBank, VectorBase (Amos et al. 2022; Giraldo-Calderón et al. 2015), and InsectBase (Mei et al. 2022) (table S6).

Phylogeny construction

To construct the phylogenomic tree, we first used BUSCO v5.4.3 to extract the amino acid sequences for the 1,436 diptera_odb10 proteins present and with only a single copy in each assembly. We removed non-homologous characters from unaligned amino acid sequences separately for each protein present in all assemblies using PREQUAL v1.02 (Whelan et al. 2018), then aligned sequences using MAFFT v7.515 (Katoh & Standley 2013). Next, we trimmed alignments using trimAl v1.4.1 (Capella-Gutiérrez et al. 2009) using heuristic selection

of the automatic method. RAxML-NG v1.1.0 (Kozlov et al. 2019) was then used to generate a maximum likelihood (ML) tree with *Musca domestica* as outgroup. We partitioned by gene and used ModelTest-NG v0.1.7 to find the best-fit substitution model (based on AIC) for each partition (Darriba et al. 2020). We assessed branch support using both Felsenstein's (1985) and transfer (Lemoine et al. 2018) bootstrap, and we used the MRE-based bootstopping test (Pattengale et al. 2010) every 50 replicates to stop bootstrapping when it converged; our bootstrapping converged after 100 replicates.

We created a time-calibrated, ultrametric tree by combining the ML tree with fossil records (queried from paleobiodb.org on 5 July 2022) using the Bayesian MCMCTree program in the PAML v4.10.6 (Yang 2007) package. Before running MCMCTree, we used (1) PAML's CODEML program to estimate the overall substitution rate and (2) the "data-driven birth–death" (ddBD) method (Tao et al. 2021) to calculate priors for the speciation birth–death process. We ran CODEML on the ML tree using three calibration point estimates, which were simplifications compared to those used in MCMCTree (described below): 232.7 million years ago (Ma) for family Chironomidae (Cranston et al. 2012), 137.573 Ma for subfamily Chironominae (Cranston et al. 2012), and 76 Ma separating genera *Belgica* and *Clunio* (timetree.org). The ddBD method was run on the tree output from RelTime-ML (Mello et al. 2017) in MEGA-CC v11.0.13-1 (Tamura et al. 2021) that took the initial ML tree and concatenated protein sequence alignments as inputs. We used the LG model (the most common best-fit model in the ML tree) and a single concatenated alignment for both CODEML and RelTime-ML.

In MCMCTree, we used an autocorrelated clock, and we used ModelFinder (Kalyaanamoorthy et al. 2017) to merge partitions and to find the best substitution model for each partition. We used ModelFinder here because MCMCTree had difficulty converging

without merging partitions, but we used ModelTest-NG for our ML tree because it generated a better-fitting tree as indicated by AIC. We ran the MCMC for 250,000 iterations with a sample frequency of 5, after 2,000 iterations of burn-in. For MCMCTree's rate-drift parameter priors, we used a shape parameter of 1 and scale parameter equal to the mean root age (Inoue et al. 2010); we approximated mean root age by using the middle point of the uniform portion of the distribution used for the priors on root age (i.e., $(t_L + t_U)\ /\ 2$ in Table S9). To check for convergence, we ran the program 4 times and calculated the effective sample size across all parameters and runs using effectiveSize in the R package coda v0.19.4 (Plummer et al. 2006). For MCMCTree's substitution rate priors, we used a shape parameter of 2.0 for a relatively diffuse prior and a rate value that caused the Gamma distribution mean to equal the substitution rate estimated by CODEML (8.7005×10^{-10} substitutions per year). We used both fossil evidence and previous time estimates (Benton et al. 2009; Hedges & Kumar 2009; Cranston et al. 2012, 2010) to inform the parameters defining the divergence time sampling distributions in MCMCTree. We defined five calibration points: (a) 238.5 Ma minimum and 295.4 Ma maximum for the root (Benton et al. 2009), (b) 242.0 Ma minimum for the superfamily Chironomoidea (Lukashevich et al. 2010), (c) 201.3 Ma minimum for family Chironomidae (Krzeminski & Jarzembowski 1999), (d) 93.5 Ma minimum for subfamily Chironominae (Giłka et al. 2022), and (e) 33.9 Ma minimum for the portion of subfamily Orthocladiinae containing genera *Belgica* and *Clunio* (Zelentsov et al. 2012). All constraints were soft, and we adjusted the probability of sampling a time that violates the constraint based on our perceived reliability of the bounds. MCMCTree samples minimum constraints with a truncated Cauchy distribution, and we defined the Cauchy parameters to make the mode near our best estimate and the variance proportional to our confidence in this estimate (see table S9 for details on parameter values).

Finding orthogroups

We used OrthoFinder v2.5.4 (Emms & Kelly 2019) to identify phylogenetic Hierarchical Orthologous Groups (HOGs) for use in the analyses for intron sizes, gene family evolution, and positive selection. Using OrthoFinder for generating HOGs and OrthoDB for the phylogeny allowed us to generate a species tree using the more highly curated OrthoDB protein sequences while identifying more orthologs that span a greater number of biological functions for our gene family and selection analyses. We first filtered all species' protein sequences to only include the longest isoform for each protein-coding gene. We input these filtered protein sets and the time-calibrated species tree from MCMCTree into OrthoFinder and used the set of HOGs for the root of the phylogeny for downstream analyses. We removed *Clunio marinus* from any analyses using HOGs because only 58.5% of its genes were assigned to an orthogroup by OrthoFinder, compared to >90% for all other species.

Features associated with genome size

We looked for associations between genome size and four genomic features in chironomids: protein-coding genes, intergenic sequences, introns, and repeat elements. We estimated the number of protein-coding genes and total intergenic sequence length from the assemblies' *.gff files, using the longest isoform for each gene for intergenic sequences. Repeat elements were extracted from all species' RepeatMasker annotation (*.out) files, and we aggregated all non-TE elements (rolling-circles, small RNA, satellites, simple repeats, and low complexity) into one category for analysis. We calculated average intron length based only on genes matching to HOGs that had all species represented and that contained < 4 genes per

species (Martín-Durán et al. 2021). We log_{10}-transformed these variables because they were highly right skewed, with intron lengths being transformed before calculating the mean.

We first tested for significant differences in each feature (including genome size) between chironomids and other dipterans to assess whether any associations likely pertain to the specific evolutionary history of chironomids. For these tests, we grouped *Culicoides sonorensis* (family Ceratopogonidae) with chironomids because exploratory plots made it clear that it shared similar genomic traits with its most recent common ancestors in our phylogeny (family Chironomidae) and because grouping *C. sonorensis* with Chironomidae increased all models' log-likelihoods compared to not grouping it with Chironomidae. We used phylogenetic linear regressions and tested for significance of a binary variable indicating whether the species belonged to families Ceratopogonidae or Chironomidae. We used the phylolm v2.6.2 (Ho & Ane 2014) package in R v4.3.2 (R Core Team 2023) and ran models using an Ornstein-Uhlenbeck process with a fixed state estimated at the root of the phylogeny. We next tested for whether each feature was correlated with genome size to ascertain whether any changes that occurred likely contributed to genome compaction in Chironomidae. We used the cor_phylo function in the R package phyr v1.1.2 (Li et al. 2020) to compute the Pearson correlation coefficient between features and genome size while accounting for phylogenetic covariance. We constrained the phylogenetic signal to be between 0 and 1 when not doing so resulted in the covariance matrix not being positive definite, which prevented bootstrapping. For both sets of analyses, we used parametric bootstrapping (2,000 repetitions) to compute 95% confidence intervals for the parameters of interest, and we scaled the phylogeny to a maximum depth of 1 for easier interpretation because α scales with phylogeny depth (Ives & Garland 2010).

Gene family evolution

We used CAFE v5.0.0 (Mendes et al. 2020) to analyze gene family evolution along our phylogeny, looking specifically for families that expanded for Chironomidae. After CAFE removed families not present at the root of the phylogeny, we analyzed 9,057 total HOGs across our 13 species. We used a Poisson root frequency distribution and a Gamma model with 8 categories; we used 8 because from testing, this is lowest value where the changes in the negative log likelihood began to subside. From the CAFE output, we filtered for HOGs that significantly expanded ($P < 0.001$) at the node separating Chironomidae from their most recent common ancestor. To better understand the functions of these expanded HOGs, we connected Gene Ontology (GO) terms from our functional annotations to each HOG based on all genes from all species that matched to it. We then used the enricher function in the R package clusterProfiler v4.8.2 (Wu et al. 2021) to find enriched GO terms in our set of HOGs, using a Benjamini–Hochberg adjusted *P*-value cutoff of 0.1 and minimum gene size of 1. Next, we used the R package rrvgo v1.12.2 (Sayols 2023)—based on the online tool REVIGO (Supek et al. 2011)—to reduce the redundancy of the set of enriched "biological process" GO terms based on the *Drosophila melanogaster*-derived database org.Dm.eg.db v3.17.0 (Carlson 2023b) and to summarize these GO terms using a treemap.

Positive selection

We used HyPhy v2.5.52 (Murrell et al. 2015; Wertheim et al. 2015) to test for positive selection in Chironomidae. We first filtered for HOGs that contained exactly one copy per species and that were associated with at least one GO term (including all offspring) from our *a priori* list of terms associated with tolerance to extreme habitats (table S5). We identified

offspring GO terms using GO.db v3.17.0 (Carlson 2023a). Because HyPhy requires codon-aware sequence alignments, we extracted the coding sequence (CDS) for the longest isoform per gene and used HyPhy's codon-msa tool (https://github.com/veg/hyphy-analyses) and MAFFT for the alignments for each HOG. For 12 HOGs, alignments failed in one or more species, so these HOGs were removed from our analyses. Next, we labelled Chironomidae on our tree using phylotree.js (Shank et al. 2018) so that we could use chironomids as the test branches (with the others being background) for our analyses. For each HOG, we tested (1) whether positive selection occurred for any chironomids using HyPhy's BUSTED method (Murrell et al. 2015) and (2) whether selection intensified or relaxed for chironomids compared to outgroups using HyPhy's RELAX method (Wertheim et al. 2015). The resulting set of *P*-values for each test across all HOGs were corrected for multiple comparisons using the Benjamini–Yekutieli procedure (Benjamini & Yekutieli 2001) under arbitrary dependence assumptions and using a false discovery rate of 0.10.

References

Abrusán G, Grundmann N, DeMester L, Makalowski W. 2009. TEclass—a tool for automated classification of unknown eukaryotic transposable elements. Bioinformatics. 25:1329–1330.

Amos B et al. 2022. VEuPathDB: the eukaryotic pathogen, vector and host bioinformatics resource center. Nucleic Acids Research. 50:D898–D911.

Aramaki T et al. 2020. KofamKOALA: KEGG Ortholog assignment based on profile HMM and adaptive score threshold. Bioinformatics. 36:2251–2252.

Bao W, Kojima KK, Kohany O. 2015. Repbase update, a database of repetitive elements in eukaryotic genomes. Mobile DNA. 6:11.

Behura SK et al. 2011. Complete sequences of mitochondria genomes of *Aedes aegypti* and *Culex quinquefasciatus* and comparative analysis of mitochondrial DNA fragments inserted in the nuclear genomes. Insect Biochemistry and Molecular Biology. 41:770–777.

Benjamini Y, Yekutieli D. 2001. The control of the false discovery rate in multiple testing under dependency. The Annals of Statistics. 29.

Benton MJ, Donoghue PCJ, Asher RJ. 2009. Calibrating and constraining molecular clocks. In: The Timetree of Life. Hedges, SB & Kumar, S, editors. Oxford University Press: New York, NY, USA pp. 35–86.

Brůna T, Hoff KJ, Lomsadze A, Stanke M, Borodovsky M. 2021. BRAKER2: automatic eukaryotic genome annotation with GeneMark-EP+ and AUGUSTUS supported by a protein database. NAR Genomics and Bioinformatics. 3:lqaa108.

Brůna T, Lomsadze A, Borodovsky M. 2020. GeneMark-EP+: eukaryotic gene prediction with self-training in the space of genes and proteins. NAR Genomics and Bioinformatics. 2:lqaa026.

Buchfink B, Reuter K, Drost H-G. 2021. Sensitive protein alignments at tree-of-life scale using DIAMOND. Nature Methods. 18:366–368.

Bushnell B. 2014. BBMap: a fast, accurate, splice-aware aligner.

Capella-Gutiérrez S, Silla-Martínez JM, Gabaldón T. 2009. trimAl: a tool for automated alignment trimming in large-scale phylogenetic analyses. Bioinformatics. 25:1972–1973.

Carlson M. 2023a. GO.db: a set of annotation maps describing the entire Gene Ontology.

Carlson M. 2023b. org.Dm.eg.db: genome wide annotation for fly.

Chakraborty M et al. 2021. Hidden genomic features of an invasive malaria vector, *Anopheles stephensi*, revealed by a chromosome-level genome assembly. BMC Biology. 19:28.

Chakraborty M, Baldwin-Brown JG, Long AD, Emerson JJ. 2016. Contiguous and accurate *de novo* assembly of metazoan genomes with modest long read coverage. Nucleic Acids Research. gkw654.

Chen S, Zhou Y, Chen Y, Gu J. 2018. fastp: an ultra-fast all-in-one FASTQ preprocessor. Bioinformatics. 34:i884–i890.

Chen Y et al. 2021. Efficient assembly of nanopore reads via highly accurate and intact error correction. Nature Communications. 12:60.

Cranston PS, Hardy NB, Morse GE. 2012. A dated molecular phylogeny for the Chironomidae (Diptera). Systematic Entomology. 37:172–188.

Cranston PS, Hardy NB, Morse GE, Puslednik L, McCluen SR. 2010. When molecules and morphology concur: the 'Gondwanan' midges (Diptera: Chironomidae). Systematic Entomology. 35:636–648.

Darriba D et al. 2020. ModelTest-NG: a new and scalable tool for the selection of dna and protein evolutionary models. Molecular Biology and Evolution. 37:291–294.

El-Gebali S et al. 2019. The Pfam protein families database in 2019. Nucleic Acids Research. 47:D427–D432.

Emms DM, Kelly S. 2019. OrthoFinder: phylogenetic orthology inference for comparative genomics. Genome Biology. 20:238.

Felsenstein J. 1985. Confidence limits on phylogenies: an approach using the bootstrap. Evolution. 39:783–791.

Flynn JM et al. 2020. RepeatModeler2 for automated genomic discovery of transposable element families. Proceedings of the National Academy of Sciences. 117:9451–9457.

Gabriel L, Hoff KJ, Brůna T, Borodovsky M, Stanke M. 2021. TSEBRA: transcript selector for BRAKER. BMC Bioinformatics. 22:566.

Giłka W et al. 2022. Wanted, tracked down and identified: Mesozoic non-biting midges of the subfamily Chironominae (Chironomidae, Diptera). Zoological Journal of the Linnean Society. 194:874–892.

Giraldo-Calderón GI et al. 2015. VectorBase: an updated bioinformatics resource for invertebrate vectors and other organisms related with human diseases. Nucleic Acids Research. 43:D707–D713.

Guan D et al. 2020. Identifying and removing haplotypic duplication in primary genome assemblies. Bioinformatics. 36:2896–2898.

Hedges SB, Kumar S, eds. 2009. *The Timetree of Life*. Oxford University Press: New York, NY, USA.

Ho LST, Ane C. 2014. A linear-time algorithm for Gaussian and non-Gaussian trait evolution models. Systematic Biology. 63:397–408.

Hu J et al. 2023. An efficient error correction and accurate assembly tool for noisy long reads. doi: 10.1101/2023.03.09.531669.

Hu J, Fan J, Sun Z, Liu S. 2020. NextPolish: a fast and efficient genome polishing tool for long-read assembly. Bioinformatics. 36:2253–2255.

Huerta-Cepas J et al. 2019. eggNOG 5.0: a hierarchical, functionally and phylogenetically annotated orthology resource based on 5090 organisms and 2502 viruses. Nucleic Acids Research. 47:D309–D314.

Inoue J, Donoghue PCJ, Yang Z. 2010. The impact of the representation of fossil calibrations on Bayesian estimation of species divergence times. Systematic Biology. 59:74–89.

Ives AR, Garland T. 2010. Phylogenetic logistic regression for binary dependent variables. Systematic Biology. 59:9–26.

Kaiser TS et al. 2016. The genomic basis of circadian and circalunar timing adaptations in a midge. Nature. 540:69–73.

Kalyaanamoorthy S, Minh BQ, Wong TKF, Von Haeseler A, Jermiin LS. 2017. ModelFinder: fast model selection for accurate phylogenetic estimates. Nature Methods. 14:587–589.

Katoh K, Standley DM. 2013. MAFFT Multiple Sequence Alignment Software Version 7: Improvements in Performance and Usability. Molecular Biology and Evolution. 30:772–780.

Kelley JL et al. 2014. Compact genome of the Antarctic midge is likely an adaptation to an extreme environment. Nature Communications. 5:1–8.

Kim D, Paggi JM, Park C, Bennett C, Salzberg SL. 2019. Graph-based genome alignment and genotyping with HISAT2 and HISAT-genotype. Nature Biotechnology. 37:907–915.

Kolmogorov M, Yuan J, Lin Y, Pevzner PA. 2019. Assembly of long, error-prone reads using repeat graphs. Nature Biotechnology. 37:540–546.

Kozlov AM, Darriba D, Flouri T, Morel B, Stamatakis A. 2019. RAxML-NG: a fast, scalable and user-friendly tool for maximum likelihood phylogenetic inference. Bioinformatics. 35:4453–4455.

Kriventseva EV et al. 2019. OrthoDB v10: sampling the diversity of animal, plant, fungal, protist, bacterial and viral genomes for evolutionary and functional annotations of orthologs. Nucleic Acids Research. 47:D807–D811.

Krzeminski W, Jarzembowski E. 1999. *Aenne triassica* sp. n., the oldest representative of the family Chironomidae (Insecta: Diptera). Polskie Pismo Entomologiczne. 68:445–449.

Kutsenko A et al. 2014. The *Chironomus tentans* genome sequence and the organization of the Balbiani ring genes. BMC Genomics. 15:819.

Lemoine F et al. 2018. Renewing Felsenstein's phylogenetic bootstrap in the era of big data. Nature. 556:452–456.

Li D, Dinnage R, Nell LA, Helmus MR, Ives AR. 2020. phyr: an R package for phylogenetic species-distribution modelling in ecological communities. Methods in Ecology and Evolution. 11:1455–1463.

Li H. 2013. Aligning sequence reads, clone sequences and assembly contigs with BWA-MEM. arXiv.

Li H. 2018. Minimap2: pairwise alignment for nucleotide sequences. Bioinformatics. 34:3094–3100.

Liu H, Wu S, Li A, Ruan J. 2021. SMARTdenovo: a de novo assembler using long noisy reads. Gigabyte. 2021:1–9.

Lomsadze A, Burns PD, Borodovsky M. 2014. Integration of mapped RNA-Seq reads into automatic training of eukaryotic gene finding algorithm. Nucleic Acids Research. 42:e119–e119.

Lu S et al. 2020. CDD/SPARCLE: the conserved domain database in 2020. Nucleic Acids Research. 48:D265–D268.

Lukashevich ED, Przhiboro AA, Marchal-Papier F, Grauvogel-Stamm L. 2010. The oldest occurrence of immature Diptera (Insecta), Middle Triassic, France. Annales de la Société Entomologique de France. 46:4–22.

MacManes MD. 2014. On the optimal trimming of high-throughput mRNA sequence data. Frontiers in Genetics. 5.

Manni M, Berkeley MR, Seppey M, Simão FA, Zdobnov EM. 2021. BUSCO update: novel and streamlined workflows along with broader and deeper phylogenetic coverage for scoring of eukaryotic, prokaryotic, and viral genomes. Molecular Biology and Evolution. 38:4647–4654.

Martín-Durán JM et al. 2021. Conservative route to genome compaction in a miniature annelid. Nature Ecology & Evolution. 5:231–242.

Matthews BJ et al. 2018. Improved reference genome of *Aedes aegypti* informs arbovirus vector control. Nature. 563:501–507.

Mei Y et al. 2022. InsectBase 2.0: a comprehensive gene resource for insects. Nucleic Acids Research. 50:D1040–D1045.

Mello B, Tao Q, Tamura K, Kumar S. 2017. Fast and accurate estimates of divergence times from big data. Molecular Biology and Evolution. 34:45–50.

Mendes FK, Vanderpool D, Fulton B, Hahn MW. 2020. CAFE 5 models variation in evolutionary rates among gene families. Bioinformatics. 36:5516–5518.

Morales-Hojas R et al. 2018. The genome of the biting midge *Culicoides sonorensis* and gene expression analyses of vector competence for Bluetongue virus. BMC Genomics. 19:624.

Murrell B et al. 2015. Gene-wide identification of episodic selection. Molecular Biology and Evolution. 32:1365–1371.

Pattengale ND, Alipour M, Bininda-Emonds ORP, Moret BME, Stamatakis A. 2010. How many bootstrap replicates are necessary? Journal of Computational Biology. 17:337–354.

Pfenninger M, Schönnenbeck P, Schell T. 2022. ModEst: Accurate estimation of genome size from next generation sequencing data. Molecular Ecology Resources. 22:1454–1464.

Plummer M, Best N, Cowles K, Vines K. 2006. CODA: convergence diagnosis and output analysis for MCMC. R News. 6:7–11.

Queirós P, Delogu F, Hickl O, May P, Wilmes P. 2021. Mantis: flexible and consensus-driven genome annotation. GigaScience. 10:giab042.

Quick J. 2018. Ultra-long read sequencing protocol for RAD004 v3. doi: 10.17504/protocols.io.mrxc57n.

R Core Team. 2023. R: A Language and Environment for Statistical Computing. https://www.r-project.org/.

Saier MH et al. 2021. The Transporter Classification Database (TCDB): 2021 update. Nucleic Acids Research. 49:D461–D467.

Sayols S. 2023. rrvgo: a Bioconductor package for interpreting lists of Gene Ontology terms. microPublication Biology.

Scott JG et al. 2014. Genome of the house fly, *Musca domestica* L., a global vector of diseases with adaptations to a septic environment. Genome Biology. 15:466.

Shaikhutdinov NM et al. 2023. Population genomics of two closely related anhydrobiotic midges reveals differences in adaptation to extreme desiccation. Genome Biology and Evolution. 15:evad169.

Shank SD, Weaver S, Kosakovsky Pond SL. 2018. phylotree.js - a JavaScript library for application development and interactive data visualization in phylogenetics. BMC Bioinformatics. 19:276.

Shin SC et al. 2019. Nanopore sequencing reads improve assembly and gene annotation of the *Parochlus steinenii* genome. Scientific reports. 9:1–10.

Smit A, Hubley R, Green P. 2013. *RepeatMasker Open-4.0*. http://www.repeatmasker.org.

Stanke M, Diekhans M, Baertsch R, Haussler D. 2008. Using native and syntenically mapped cDNA alignments to improve de novo gene finding. Bioinformatics. 24:637–644.

Steward RA, Okamura Y, Boggs CL, Vogel H, Wheat CW. 2021. The genome of the margined white butterfly (*Pieris macdunnoughii*): sex chromosome insights and the power of polishing with PoolSeq data. Genome Biology and Evolution. 13:evab053.

Sun X et al. 2021. A chromosome level genome assembly of *Propsilocerus akamusi* to understand its response to heavy metal exposure. Molecular Ecology Resources. 21:1996–2012.

Supek F, Bošnjak M, Škunca N, Šmuc T. 2011. REVIGO summarizes and visualizes long lists of Gene Ontology terms. PLoS ONE. 6:e21800.

Tamura K, Stecher G, Kumar S. 2021. MEGA11: molecular evolutionary genetics analysis version 11. Molecular Biology and Evolution. 38:3022–3027.

Tao Q, Barba-Montoya J, Kumar S. 2021. Data-driven speciation tree prior for better species divergence times in calibration-poor molecular phylogenies. Bioinformatics. 37:i102–i110.

Vaser R, Sović I, Nagarajan N, Šikić M. 2017. Fast and accurate de novo genome assembly from long uncorrected reads. Genome Research. 27:737–746.

Wertheim JO, Murrell B, Smith MD, Kosakovsky Pond SL, Scheffler K. 2015. RELAX: detecting relaxed selection in a phylogenetic framework. Molecular Biology and Evolution. 32:820–832.

Whelan S, Irisarri I, Burki F. 2018. PREQUAL: detecting non-homologous characters in sets of unaligned homologous sequences. Bioinformatics.

Wu T et al. 2021. clusterProfiler 4.0: a universal enrichment tool for interpreting omics data. The Innovation. 2:100141.

Yang Z. 2007. PAML 4: phylogenetic analysis by maximum likelihood. Molecular Biology and Evolution. 24:1586–1591.

Yoshida Y et al. 2022. High quality genome assembly of the anhydrobiotic midge provides insights on a single chromosome-based emergence of extreme desiccation tolerance. NAR Genomics and Bioinformatics. 4:lqac029.

Zelentsov NI, Baranov VA, Perkovsky EE, Shobanov NA. 2012. First records on non-biting midges (Diptera: Chironomidae) from the Rovno amber. Russian Entomological Journal. 21:79–87.

Supplementary Figures and Tables

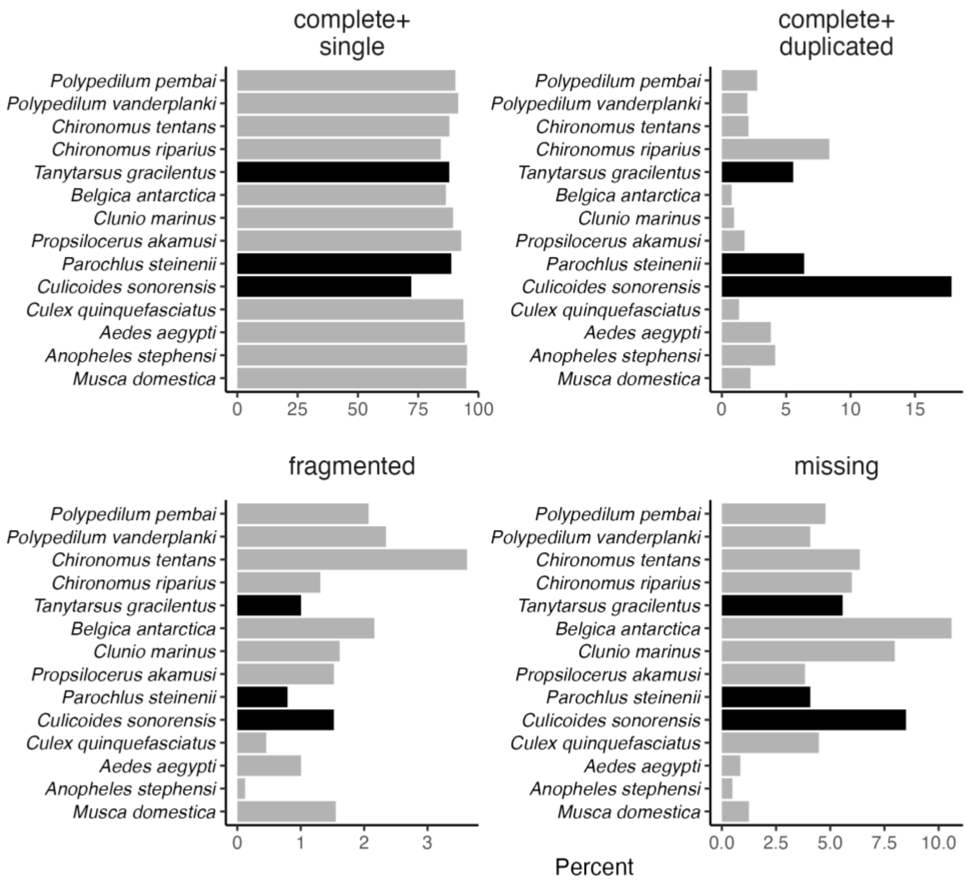

Figure S1. Transcriptome BUSCO completeness for all species our phylogeny. Species are ordered as they appear in the phylogeny (figs. 1A, S2), and the three species whose gene predictions were created here are shown in black.

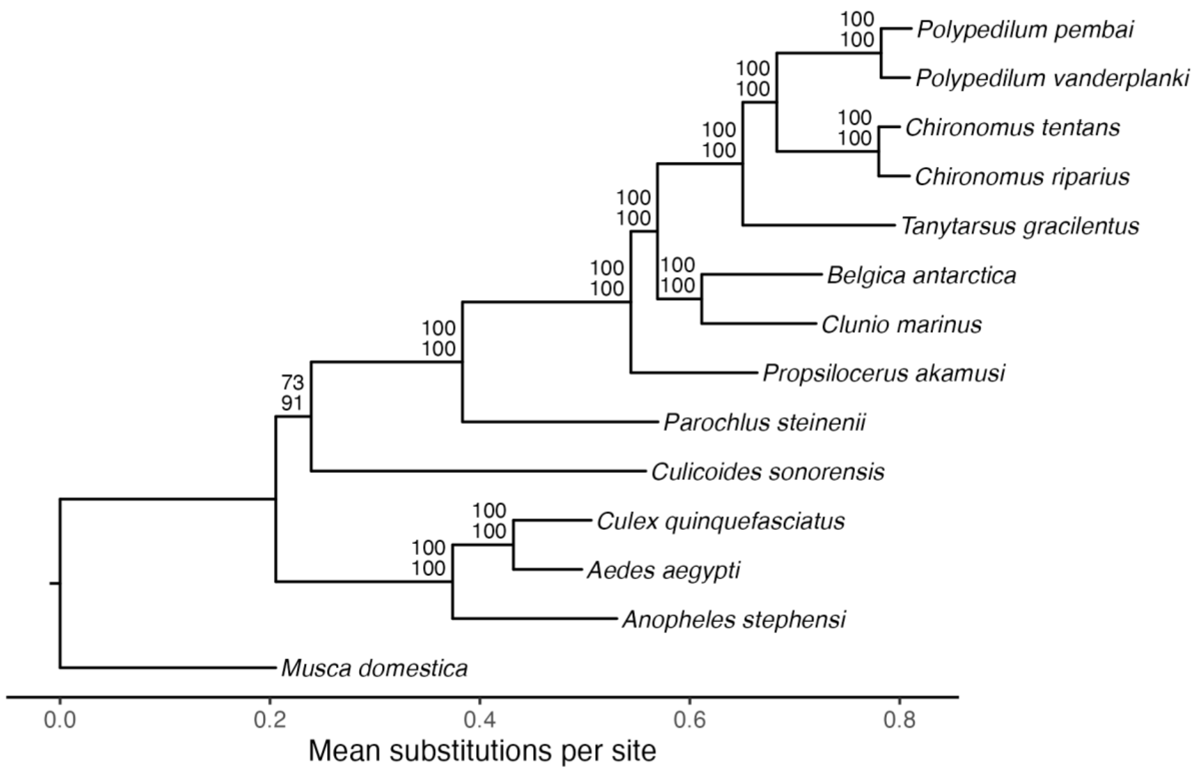

Figure S2. Maximum likelihood phylogenomic tree showing relationships among chironomids and outgroups within the order Diptera. Numbers near nodes indicate percent support using Felsenstein's bootstrap (top) and transfer bootstrap (bottom).

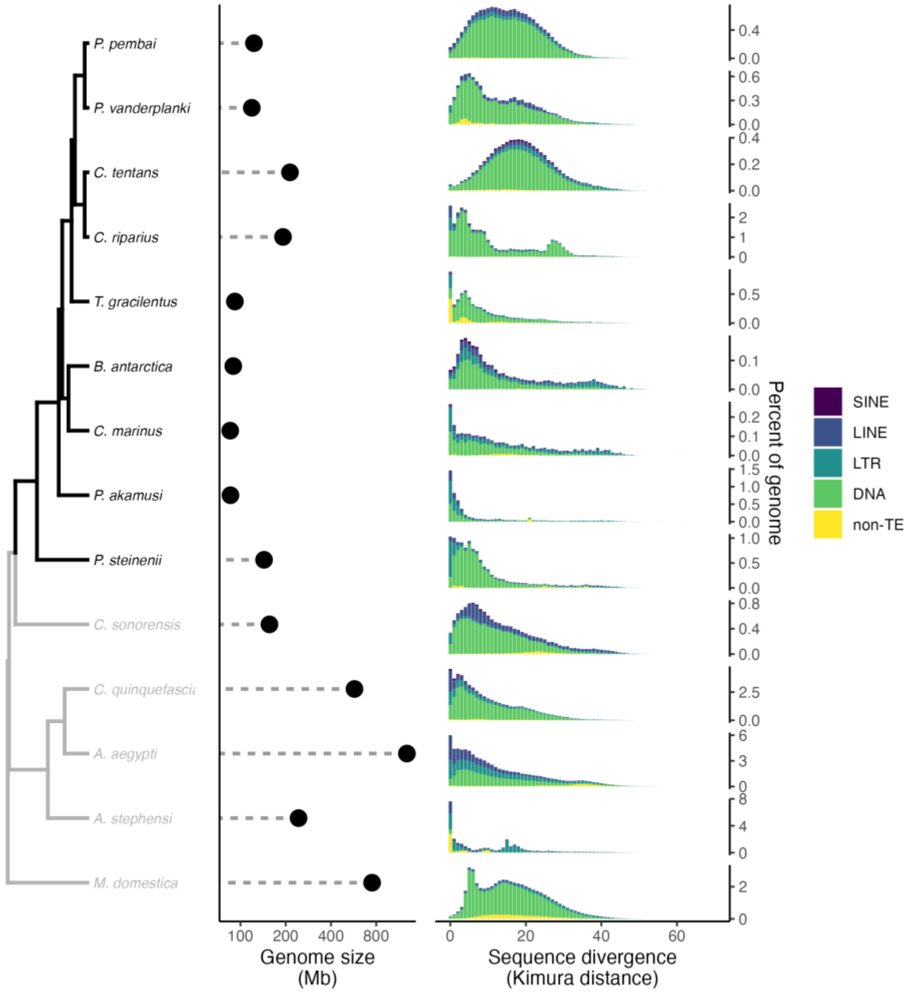

Figure S3. Distributions of repeat element divergences calculated using the CpG-adjusted Kimura substitution model (right panel) for all species in our phylogeny, where color indicates the repeat class. The phylogeny (left panel) has chironomids in black and others in gray.

A

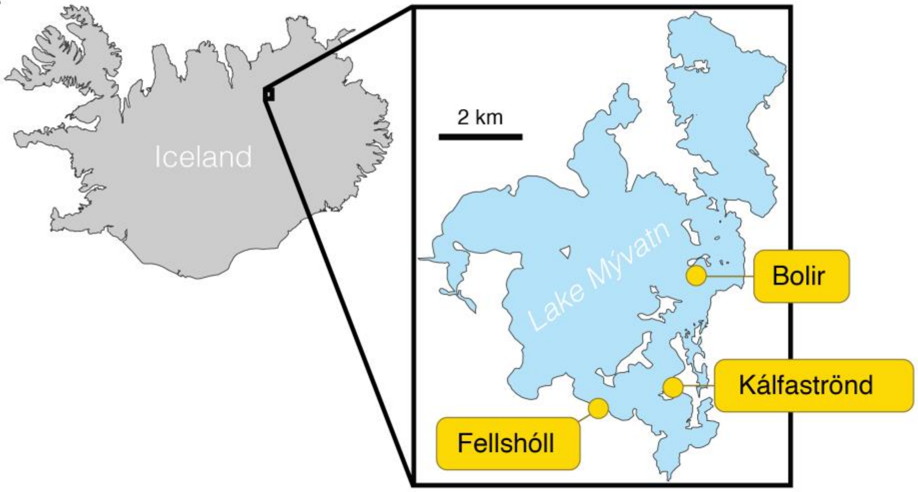

B

Individuals	Life stage	Date collected	Location	Reads type	Platform*	Use(s)
1	adult	10 Jun 2018	Fellshóll	long DNA	ONT	assembly & polishing
7	adult	21 Jun 2019	Kálfaströnd	2×150bp DNA	Illumina	polishing
10	adult	9 Aug 2021	Kálfaströnd	2×150bp RNA	Illumina	annotation
155	juvenile	12–13 Aug 2018	Bolir	2×150bp RNA	Illumina	annotation

* "ONT" = Oxford Nanopore MinION II and "Illumina" = Illumina NovaSeq 6000

Figure S4. Map and descriptions of sequenced samples. (A) Sampled *Tanytarsus gracilentus* were collected from one of three locations at Lake Mývatn, Iceland. (B) Descriptions of the four sequencing libraries used in this study, including number of *T. gracilentus* individuals in the pool, life stage of the individuals, date collected, location of collection (names coincide with part A), type of reads (long vs 2×150bp, DNA vs RNA), sequencing platform, and step(s) for which they were used.

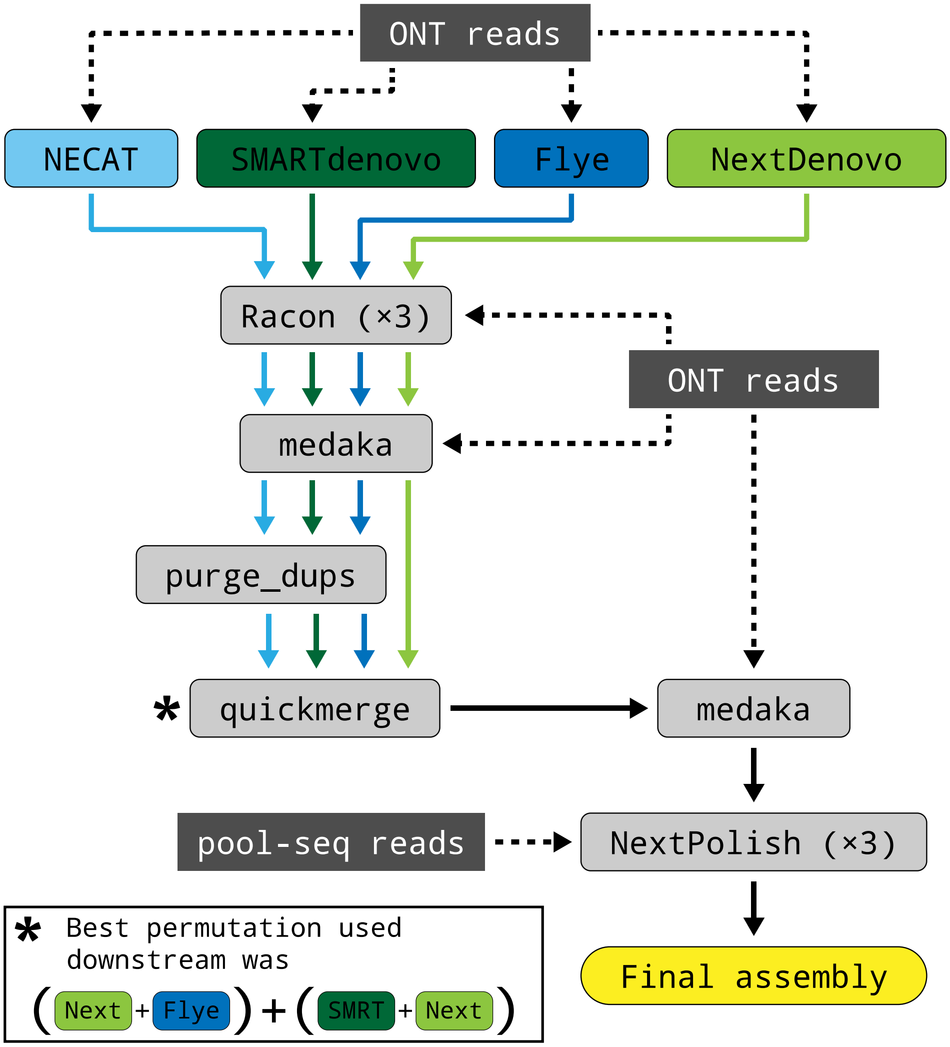

Figure S5. Pipeline to generate the *Tanytarsus gracilentus* reference assembly.

Table S1. Summary statistics for genome annotations. Included is completeness of coding sequences (CDS) calculated using BUSCO and the diptera_odb10 protein database. Also included are the number of genes with tags for Kyoto Encyclopedia of Genes and Genomes (KEGG) Orthology (KO) terms, Gene Ontology (GO) terms, Clusters of Orthologous Genes (COG) categories, Enzyme Commission (EC) numbers, and KEGG enzyme pathways.

Feature	Assembly			
	*Chironomus riparius**	*Culicoides sonorensis*	*Parochlus steinenii*	*Tanytarsus gracilentus*
total proteins	–	18,212	16,259	15,561
protein-coding genes	16,522	18,080	16,105	15,499
% CDS complete	–	90.0	95.1	93.4
% CDS complete + single	–	72.1	88.7	87.9
% CDS complete + duplicated	–	17.8	6.4	5.5
KO terms	10,576	10,760	9,675	9,753
GO terms	7,414	7,907	7,095	6,834
COG categories	3,937	4,411	4,009	3,785
EC numbers	3,698	3,870	3,517	3,566
KEGG enzyme pathways	3,080	3,427	3,074	2,927

* This species only had functional annotation created here so did not have CDS completeness scored

Table S2. Summary statistics for BUSCO matching and resulting amino acid sequence alignments. Shown is the number of proteins from OrthoDB's diptera_odb10 database that match a protein in the assembly of each species (with exactly one copy). Also shown is the percent of missing data (before trimming) in each species' protein alignments across all 1,437 genes that were present and single-copy in all species.

Species	Matching genes	Alignment missing (%)
Chironomus tentans	2,880	23.21
Chironomus riparius	2,982	21.56
Polypedilum vanderplanki	3,002	21.14
Polypedilum pembai	2,932	22.95
Tanytarsus gracilentus	2,973	22.58
Belgica antarctica	2,990	22.09
Clunio marinus	3,022	20.31
Propsilocerus akamusi	2,997	21.82
Parochlus steinenii	2,986	26.25
Culicoides sonorensis	2,501	28.45
Culex quinquefasciatus	3,062	21.90
Aedes aegypti	3,071	22.28
Anopheles stephensi	3,073	17.89
Musca domestica	3,111	23.53

Table S3. Estimates and parametric-bootstrapped 95% confidence interval bounds for Pearson correlations between genome size and genome features computed using cor_phylo.

	Feature	r	$r_{2.5\%}$	$r_{97.5\%}$
	Protein-coding genes	0.218	−0.322	0.627
	Intergenic content	0.952	0.639	1.000
	Mean intron length	0.866	0.500	0.999
Repeat elements	DNA	0.789	0.431	0.951
	LINE	0.676	0.240	0.908
	LTR	0.727	0.312	0.922
	SINE	0.721	0.271	0.969
	Unclassified	0.783	0.420	0.949
	Non-TE	0.710	0.270	0.972

Table S4. List of the nine HOG gene families that significantly expanded at the node separating Chironomidae from their most recent common ancestor in our phylogeny. Listed are the gene(s) for *Culex quinquefasciatus* (Cquinq) and *Musca domestica* (Mdomes) that fall within the HOG and the descriptions for these genes from each species' genome annotation.

HOG	Species	Gene	Description
N0.HOG0000131	Cquinq	CQUJHB001249	facilitated trehalose transporter Tret1-like
	Cquinq	CQUJHB005468	facilitated trehalose transporter Tret1-like
	Cquinq	CQUJHB010329	facilitated trehalose transporter Tret1
	Mdomes	Mdom004618	Facilitated trehalose transporter Tret1-2 homolog
N0.HOG0000001	Cquinq	CQUJHB008986	cytochrome P450 6a14
	Cquinq	CQUJHB009005	cytochrome P450 6A1
	Cquinq	CQUJHB009148	cytochrome P450 6a14
	Mdomes	Mdom002048	cytochrome P450 317a1
N0.HOG0000230	Cquinq	CQUJHB000177	serine protease gd
	Cquinq	CQUJHB000414	serine protease gd-like
	Cquinq	CQUJHB001030	CLIP domain-containing serine protease B15
	Cquinq	CQUJHB004853	coagulation factor IX-like
	Cquinq	CQUJHB005641	serine protease gd
	Cquinq	CQUJHB008470	chymotrypsin-like protease CTRL-1
	Cquinq	CQUJHB013601	transmembrane protease serine 12-like
	Mdomes	Mdom003072	Serine protease gd
N0.HOG0000162	Cquinq	CQUJHB000433	N-acetylgalactosaminyltransferase 6
	Cquinq	CQUJHB009199	N-acetylgalactosaminyltransferase 6
	Mdomes	Mdom000331	N-acetylgalactosaminyltransferase 6
	Mdomes	Mdom003911	N-acetylgalactosaminyltransferase 4
	Mdomes	Mdom011216	N-acetylgalactosaminyltransferase 6
	Mdomes	Mdom012602	N-acetylgalactosaminyltransferase 4
N0.HOG0000258	Cquinq	CQUJHB000140	putative helicase mov-10-B.1
	Cquinq	CQUJHB000475	putative helicase mov-10-B.1
	Cquinq	CQUJHB001205	putative helicase mov-10-B.1
	Cquinq	CQUJHB002398	putative helicase MOV-10
	Cquinq	CQUJHB004211	putative helicase MOV-10
	Cquinq	CQUJHB006152	putative helicase mov-10-B.1
	Cquinq	CQUJHB011488	putative helicase MOV-10
	Cquinq	CQUJHB011735	putative helicase MOV-10
	Cquinq	CQUJHB012713	putative helicase MOV-10
	Cquinq	CQUJHB013282	putative helicase MOV-10
	Cquinq	CQUJHB015355	putative helicase MOV-10
	Mdomes	Mdom001916	Putative helicase mov-10-B.1
	Mdomes	Mdom001919	Putative helicase mov-10-B.1
	Mdomes	Mdom010470	Putative helicase mov-10-B.1

N0.HOG0000213	Mdomes	Mdom011928	Microsomal glutathione S-transferase 1
	Mdomes	Mdom012368	Microsomal glutathione S-transferase 1
N0.HOG0000320	Mdomes	Mdom013520	ATP-binding cassette sub-family G member 1
N0.HOG0000559	Cquinq	CQUJHB009847	glycoprotein hormone G-protein coupled receptor
	Mdomes	Mdom011711	Adenosine receptor A2a
N0.HOG0000304	Mdomes	Mdom003891	Cathepsin L
	Mdomes	Mdom012445	Cathepsin L

Table S5. Targeted Gene Ontology (GO) terms used in the analyses for positive selection.

GO term	Description	Parent category
GO:0001666	response to hypoxia	response to stimulus ><br>response to stress
GO:0010038	response to metal ion	response to stimulus ><br>response to chemical ><br>response to inorganic substance
GO:0098542	defense response to other organism	response to stimulus ><br>response to external stimulus ><br>response to stress ><br>response to other organism
GO:0006979	response to oxidative stress	response to stimulus ><br>response to stress
GO:0034059	response to anoxia	response to stimulus ><br>response to stress
GO:0009408	response to heat	response to stimulus ><br>response to stress
GO:0010212	response to ionizing radiation	response to stimulus ><br>response to abiotic stimulus ><br>response to radiation
GO:0009409	response to cold	response to stimulus ><br>response to stress

Table S6. Sources of all genome assemblies and annotations used in this study. Unless otherwise noted, each annotation source provides a set of both gene predictions and functional annotations with GO terms. Families are abbreviated as follows: Ch = Chironomidae, Ce = Ceratopogonidae, Cu = Culicidae, and Mu = Muscidae.

		Assembly		Annotation	
Family	Species	Source	Accession	Source	Accession
Ch	*Tanytarsus gracilentus*	Present study	–	Present study	–
	Chironomus riparius	Rothamsted Research	GCA_917627325.3	GenBank*	GCA_917627325.3
	Chironomus tentans	(Kutsenko et al. 2014)	GCA_000786525.1	InsectBase	IBG_00179
	Polypedilum vanderplanki	(Yoshida et al. 2022)	GCA_018290095.1	InsectBase	IBG_00656
	Polypedilum pembai	(Shaikhutdinov et al. 2023)	GCA_014622435.1	InsectBase	IBG_00655
	Belgica antarctica	(Kelley et al. 2014)	GCA_000775305.1	InsectBase	IBG_00108
	Clunio marinus	(Kaiser et al. 2016)	GCA_900005825.1	InsectBase	IBG_00191
	Propsilocerus akamusi	(Sun et al. 2021)	GCA_018397935.1	InsectBase	IBG_00659
	Parochlus steinenii	Present study†	–	Present study	–
Ce	*Culicoides sonorensis*	(Morales-Hojas et al. 2018)	GCA_900258525.3	Present study	–
Cu	*Anopheles stephensi*	(Chakraborty et al. 2021)	GCA_013141755.1	VectorBase	GCF_013141755.1, Sep 08, 2020
	Aedes aegypti	(Matthews et al. 2018)	GCA_002204515.1	VectorBase	GCA_002204515.1, AaegL5.3
	Culex quinquefasciatus	(Behura et al. 2011)	GCA_015732765.1	VectorBase	GCA_015732765.1, Dec 04, 2020
Mu	*Musca domestica*	(Scott et al. 2014)	GCA_000371365.1	InsectBase	IBG_00558

* Functional annotation with GO terms was not found on GenBank, so it was generated in this study

† Based on reads from (Shin et al. 2019)

Table S7. Summary statistics for *Tanytarsus gracilentus* assemblies. In the "program" column, ">" indicates that this program was run on the output from the previous step, and the quickmerge assembly shown is the best permutation (see table S7).

program	size (Mb)	# contigs	N50 (Mb)	min (bp)	max (Mb)	% BUSCOs (n = 3,285)
NECAT	93.49	198	2.15	619	3.88	C:88.65 [S:86.61, D:2.04], F:2.68, M:8.68
> Racon	93.60	192	2.16	1,062	3.90	C:89.28 [S:87.28, D:2.01], F:2.62, M:8.10
> medaka	93.85	192	2.17	1,070	3.91	C:90.20 [S:88.10, D:2.10], F:1.83, M:7.98
> purge_dups	91.90	121	2.13	2,004	3.91	C:90.20 [S:88.68, D:1.52], F:1.80, M:8.01
SMARTdenovo	95.11	159	1.40	7,167	5.34	C:87.67 [S:85.18, D:2.50], F:3.53, M:8.80
> Racon	95.42	159	1.41	5,040	5.37	C:89.77 [S:87.25, D:2.53], F:2.65, M:7.58
> medaka	95.47	159	1.41	5,077	5.37	C:90.96 [S:88.22, D:2.74], F:1.77, M:7.28
> purge_dups	92.84	119	1.52	6,612	5.37	C:90.87 [S:89.19, D:1.67], F:1.80, M:7.34
NextDenovo	93.03	88	2.23	16,933	6.77	C:89.65 [S:88.46, D:1.19], F:2.34, M:8.01
> Racon	92.80	88	2.23	16,037	6.76	C:89.80 [S:88.80, D:1.00], F:2.47, M:7.73
> medaka	92.61	88	2.23	16,049	6.76	C:90.93 [S:89.95, D:0.97], F:1.74, M:7.34
Flye	97.11	1,114	0.93	495	3.70	C:89.68 [S:87.06, D:2.62], F:2.44, M:7.88
> Racon	97.51	1,052	0.94	92	3.72	C:89.71 [S:87.31, D:2.40], F:2.44, M:7.85
> medaka	97.87	1,052	0.94	92	3.74	C:90.93 [S:88.49, D:2.44], F:1.80, M:7.28
> purge_dups	91.89	333	0.99	203	3.74	C:90.93 [S:89.68, D:1.25], F:1.80, M:7.28
quickmerge	92.33	45	7.05	16,049	12.43	C:91.05 [S:90.08, D:0.97], F:1.74, M:7.21
> medaka	92.03	45	7.02	16,040	12.40	C:91.11 [S:90.20, D:0.91], F:1.74, M:7.15
> NextPolish	91.83	45	7.01	16,039	12.37	C:91.60 [S:90.53, D:1.07], F:1.49, M:6.91

Table S8. Summary statistics for two rounds of quickmerge on initial *T. gracilentus* assemblies. The permutation column indicates the identity and order of assemblies input to quickmerge, and the lower set of permutations are the second round that use the four best first-round permutations.

	permutation	size (Mb)	# contigs	N50 (Mb)	min (bp)	max (Mb)	% BUSCOs
	flye + necat	91.45	180	6.05	203	13.96	C:89.95 [S:88.65, D:1.31], F:1.80, M:8.25
	flye + next	93.32	156	6.50	203	18.17	C:90.41 [S:89.13, D:1.28], F:1.67, M:7.91
	flye + smart	89.87	192	4.50	203	11.07	C:88.19 [S:86.79, D:1.40], F:1.61, M:10.20
	necat + flye	91.45	103	2.76	2,004	13.92	C:90.11 [S:88.65, D:1.46], F:1.74, M:8.16
	necat + next	93.77	106	2.77	2,004	6.76	C:90.05 [S:86.70, D:3.35], F:1.77, M:8.19
	necat + smart	92.41	96	3.07	2,004	13.30	C:90.29 [S:88.25, D:2.04], F:1.70, M:8.01
*	next + flye	92.34	68	3.31	16,049	12.43	C:91.02 [S:90.05, D:0.97], F:1.74, M:7.25
	next + necat	93.49	75	2.89	17,544	6.76	C:90.08 [S:87.64, D:2.44], F:1.77, M:8.16
*	next + smart	92.45	62	3.31	17,544	9.01	C:90.96 [S:90.05, D:0.91], F:1.77, M:7.28
	smart + flye	92.33	69	6.30	6,612	11.25	C:90.87 [S:89.13, D:1.74], F:1.77, M:7.37
*	smart + necat	92.47	53	5.31	6,612	13.24	C:89.53 [S:87.00, D:2.53], F:1.74, M:8.74
*	smart + next	92.11	56	5.38	6,612	11.63	C:90.93 [S:89.92, D:1.00], F:1.70, M:7.37
	(next + flye) + (next + smart)	94.80	60	6.05	16,049	12.43	C:91.02 [S:87.61, D:3.41], F:1.74, M:7.25
	(next + smart) + (next + flye)	92.29	54	6.05	17,544	12.43	C:91.02 [S:90.08, D:0.94], F:1.74, M:7.25
	(next + flye) + (smart + necat)	93.06	40	7.05	17,544	13.25	C:90.62 [S:88.34, D:2.28], F:1.80, M:7.58
	(smart + necat) + (next + flye)	90.21	41	8.58	6,612	14.25	C:88.49 [S:86.88, D:1.61], F:1.70, M:9.80
†	(next + flye) + (smart + next)	92.33	45	7.05	16,049	12.43	C:91.05 [S:90.08, D:0.97], F:1.74, M:7.21
	(smart + next) + (next + flye)	92.01	46	7.05	6,612	12.43	C:90.96 [S:89.89, D:1.07], F:1.67, M:7.37

* first-round permutation used in second round
† best second-round permutation used as final quickmerge assembly

Table S9. Parameters defining the statistical distributions used to sample divergence times in MCMCTree, for calibrations defining (A) lower and upper and (B) just lower bounds. All values use 100 million years ago as the time unit because MCMCTree requires that all times be 0.01–10. (A) Parameter t_L is the lower age bound, t_U is the upper age bound, p_L is the probability that the real age is less than t_L, and p_U is the probability that the real age is greater than t_U. Upper and lower constraint specifications take the form 'B(t_L, t_U, p_L, p_U)' in MCMCTree. (B) Parameter p is the offset, c is the scale parameter. Lower constraint commands take the form 'L(t_L, p, c, p_L)'. In the Cauchy distribution used for sampling, the location parameter is $t_L[1 + p]$, and the scale parameter is $c\ t_L$. When a distribution mode was based on a reference, this means that we adjusted parameter p so that the distribution mode matched the estimate in that reference.

A. Lower and upper constraints:					
Node	t_L	t_U	p_L	p_U	Notes
a	2.385	2.954	0.025	0.1	Bounds based on (Benton et al. 2009), increased softness due to estimates in (Cranston et al. 2012)
B. Just lower constraints:					
Node	t_L	p	c	p_L	Notes
b	2.420	0.1	0.2	0.1	increased softness and a low distribution mean because of lower estimate in (Benton et al. 2009)
c	2.013	0.16	0.5	0.025	distribution mode based on (Cranston et al. 2012)
d	0.935	0.47	0.5	0.025	distribution mode based on (Cranston et al. 2012)
e	0.339	1.24	1.0	0.025	distribution mode based on (Benton et al. 2009)
